# Conformable Electrode Arrays for Wearable Neuroprostheses

**DOI:** 10.3390/s23062982

**Published:** 2023-03-09

**Authors:** Narrendar RaviChandran, Mei Ying Teo, Andrew McDaid, Kean Aw

**Affiliations:** 1 Medical Devices and Technologies Group, Department of Mechanical and Mechatronics Engineering, The University of Auckland, Auckland 1010, New Zealand; 2 Singapore Eye Research Institute, Singapore 169856, Singapore; 3 Smart Materials and Microtechnologies Group, Department of Mechanical and Mechatronics Engineering, The University of Auckland, Auckland 1010, New Zealand

**Keywords:** functional electrical stimulation, hand function, electrode array, carbon black, elastomer

## Abstract

Wearable electrode arrays can selectively stimulate muscle groups by modulating their shape, size, and position over a targeted region. They can potentially revolutionize personalized rehabilitation by being noninvasive and allowing easy donning and doffing. Nevertheless, users should feel comfortable using such arrays, as they are typically worn for an extended time period. Additionally, to deliver safe and selective stimulation, these arrays must be tailored to a user’s physiology. Fabricating customizable electrode arrays needs a rapid and economical technique that accommodates scalability. By leveraging a multilayer screen-printing technique, this study aims to develop personalizable electrode arrays by embedding conductive materials into silicone-based elastomers. Accordingly, the conductivity of a silicone-based elastomer was altered by adding carbonaceous material. The 1:8 and 1:9 weight ratio percentages of carbon black (CB) to elastomer achieved conductivities between 0.0021–0.0030 S cm^−1^ and were suitable for transcutaneous stimulation. Moreover, these ratios maintained their stimulation performance after several stretching cycles of up to 200%. Thus, a soft, conformable electrode array with a customizable design was demonstrated. Lastly, the efficacy of the proposed electrode arrays to stimulate hand function tasks was evaluated by in vivo experiments. The demonstration of such arrays encourages the realization of cost-effective, wearable stimulation systems for hand function restoration.

## 1. Introduction

Wearable neuroprostheses are transcutaneous stimulation systems that facilitate functional movements by eliciting muscle contraction. They are often deployed to aid people with motor deficits in regaining their lost function [1]. For such systems targeting the upper limbs, the selective activation of forearm muscles is critical to realize hand function tasks. Such activation is achieved by shaping a cathode across a subset of electrodes in an array. Thus, wearable neuroprostheses deploy electrode arrays to stimulate forearm muscles by integrating them into a sleeve [2,3]. However, such systems have limited user acceptance due to challenges in achieving selective activation (due to the complex musculature of the forearm) and poor control with transcutaneous delivery of charges [4,5]. Subject-specific factors also influence the overall stimulation outcome [6]. Nevertheless, such systems are clinically widespread, as they are easy to use and offer noninvasive means of stimulation. Thus, transcutaneous electrode technology is actively being researched to bring forth advances to improve stimulation outcomes and user acceptance.

Electrodes for transcutaneous stimulation applications predominantly fall into two categories: stratified and textile electrodes. Stratified electrodes have a multilayered construction that infuses metal electrodes into highly resistive layers with a superficial hydrogel layer [7]. Similarly, textile-knitting techniques integrate wire meshes (gauzes) into existing fabrics [8,9]. Both electrode types need regular wetting using hydrogel. Wetting improves stimulation performance by enabling good electrode–skin contact. However, extended use reduces their performance, as the hydrogel layer tends to dry [9,10]. Thus, recent advances in electrode technologies have shown interest in dry-contact electrodes, as they obviate the need for a hydrogel layer [11].

Patients prefer assistive technology that is personalizable and less cumbersome. Electrodes with bulkier profiles and poor electrode–skin contact are not ideal for being integrated into a wearable sleeve [7,8]. In addition, commercial electrodes can be far from desirable, as they are bulky and have a large form factor, thus constraining forearm movements. Moreover, lack of user integration and discomfort were the main reasons for discontinuing transcutaneous stimulation systems [6]. On the other hand, user integration can be improved by soft, conformable, and easily concealable (low form factor) electrode array sleeves that are easy for donning and doffing. A soft and conformable design ensures comfort and the ability to adhere to the skin for reliable electrode–skin contact. Although subject-specific intrinsic factors affect the stimulation outcome, electrode arrays can improve stimulation performance by allowing for customizability in terms of their design and materials [7,12].

The technological standpoints to improve existing surface stimulation technology include advances in distributed electrode design, choice of materials, and fabrication methods. Firstly, to improve user comfort, soft and conformable materials are desired. Soft materials are flexible and can provide breathability. Thus, they are comfortable on the skin for prolonged use [13,14]. In addition, conformable electrodes simplify wearability and maintain good skin contact. The material should also retain its electrical properties under stretching cycles during forearm movements [5,15,16]. Notably, elastomers, such as Ecoflex, have enhanced softness and a sticky surface. Adding conductive materials, such as carbon black (CB), can enhance their electrical properties while retaining conformability. Thus, the premise for achieving soft and conformable electrodes is to explore carbon-infused silicone elastomer composites [13]. Early designs for wearable sleeves have demonstrated the viability of integrated conductive rubber-based electrodes into an orthosis [17,18], and CB is widely used in elastomers and plastics as a filler to modify their mechanical and electrical properties. In addition, it has received special attention in the manufacture of conductive polymer composites [7,13,19]. Secondly, customizable and rapidly fabricable electrode arrays offer a cost-effective solution to home-based rehabilitation. Thus, this study favors screen printing as a fabrication technique, as the fabrication of carbon-infused elastomers seems to be a straightforward, scalable fabrication process. Moreover, screen printing of silicone-based elastomers has achieved several strides for wearable electronics [3,16,20,21,22].

Thus, this study aims to demonstrate the viability of fabricating electrode arrays with silicone-based elastomers infused with conductive materials using a scalable screen-printing process. The primary requirements for these electrodes are to deliver selective, comfortable, and safe stimulation. In addition, the infusion of conductive materials drives the required stimulation characteristics.

Accordingly, the objectives of this study include the following:

1. Characterizing the conductivity, stretchability, and surface profiles of the stimulation electrodes across different weight ratio percentages of base materials (CB and silicone-based elastomer).
2. Developing a wearable electrode-array-based sleeve utilizing carbon-based silicone elastomers that is conformable, lightweight, and easy to use, which is fabricated using a multilayered screen-printing process.
3. Demonstrating the efficacy of the electrodes to stimulate hand function tasks selectively and comfortably, as evaluated by in vivo experiments.

## 2. Methods

This study aimed to customize the properties of an electrode array by infusing conductive materials into silicone-based elastomers. Thus, a systematic characterization of the electrical and mechanical properties was conducted on elastomer samples infused with different weight ratio percentages of conductive material. Later, a suitable weight ratio percentage was identified and used for screen printing electrode-array-based sleeves. Additionally, the design-specific parameters of the array were derived from a model-based analysis. Lastly, the viability of the electrode array to evoke hand function tasks was assessed using in vivo experimentation.

### 2.1. Electrode Array Design

The electrode array had 24 individual electrodes arranged as 8 × 3 elements in a trapezoidal fashion. Such an arrangement was considered to target all motor point clusters of the forearm muscles [5]. Each electrode had a surface area of 150 mm^2^ with a circular geometry and a thickness of 0.5 mm. The electrode-array-based sleeve was 256 mm long and 125 and 162 mm wide across the distal (styloid process) and proximal ends (epicondylar region), respectively. The dimensions of the sleeve were considered based on forearm anthropometry. The interelectrode distance (center-to-center) between individual electrodes varied between 30 and 60 mm.

### 2.2. Model-Based Analysis

The design-specific parameters of the array, including the electrode shape, size, material properties, and thickness, could influence overall stimulation performance. Thus, a computational model was used to evaluate these parameters by studying current density and field distribution profiles under transcutaneous stimulation. These parameters were evaluated based on established metrics, such as surface-to-volume ratio (SA:V), motor threshold, and nonuniformity coefficients that quantified stimulation selectivity, comfort, and safety. Details of the computational model and metrics can be found here [12].

### 2.3. Materials and Preparation

The primary requirement for the electrode array was to comfortably attach to the forearm surface with very low interfacial stress. Accordingly, silicone-based elastomers can have good skin contact when used as a stretchable medium. In addition, their soft texture is comfortable for prolonged use. Additionally, users can personalize these elastomers to suit their skin or garments by color pigmentation. Ecoflex rubbers are platinum-catalyzed silicones that are versatile and easy to use among these elastomers. They have been widely used for prostheses and orthopedic cushioning [13,19]. Ecoflex^TM^ 00-30 (Smooth-on Inc., Macungie, PA, USA) was chosen for this study. It had a tensile strength of 200 psi, a shore A hardness of 30, and an elongation break at 900%. In addition, with a viscosity of 3000 cps, it was suitable for screen printing thin layers.

Having a high surface-to-volume ratio, CB has high adhesion to silicone surfaces. Thus, it is widely used as a conductive filler in elastomeric matrices to alter their electrical properties. It is often deployed for sensing applications [13,19]. The powdered form of CB from Vulcan^®^ (Fuel Cell Store, Bryan, TX, USA), XC72R, was used for this study. With an average particle size of 50 nm and a surface area of 245 m^2^ g^−1^, it was reported to have a conductivity of 4.5 S cm^−^^1^ [23].

The base mixture for the electrode was a carbon-infused silicone elastomer that blended a conductive material into a stretchable medium at a desired weight ratio percentage. Ecoflex^TM^ 00-30 was a two-part system. For the elastomer premix, equal parts of the two components, Part A and Part B, at a wt percentage of 1:1, were mixed at room temperature. Later, CB was infused into this elastomer premix. This infusion was performed in a pressurized glove box to prevent the agglomeration of CB into lumps. Additionally, a planetary centrifuge mixer (Mazerustar KK-50S) homogenized the uncured mixture, which was later degassed.

### 2.4. Electrode Material Characterization

Transcutaneous stimulation electrodes reportedly have a wide range of conductivity, from 3.3 × 10^−6^ to 0.01 S/cm [14,24]. Moreover, forearm pronation tends to displace motor points by 200%. Thus, the characterization exercise was to identify a suitable weight ratio percentage of the CB:Ecoflex composite that (1) had suitable conductivity, (2) could be screen printed, and (3) could conform while retaining its electrical properties during stretching.

Weight ratio percentages from 1:1 to 1:13 were tested empirically. Amongst them, 1:8, 1:9, 1:10, and 1:11 were found suitable for screen printing and underwent further characterization. The samples were characterized for conductivity, resistance change while stretching, surface profilometry, and surface morphology via scanning electron microscope (SEM) imaging. Lastly, the electrode array was fabricated with the weight ratio % that offered the best performance.

To observe the surface morphology, all the samples for SEM imaging were pre-deposited with gold to improve the visibility of grain size at higher magnification levels. Additionally, the roughness profiles of these samples were evaluated using Stylus profilometry, Bruker Dektak XT (Bruker Corporation, Billerica, MA, USA).

Conductivity measurements were conducted by measuring the sheet resistance of electrode samples with a four-point probe using a Keysight b2902a precision source/measure unit (Keysight Technologies, Santa Rosa, CA, USA). Each weight ratio percentage was characterized as a 20 × 20 mm sample. In addition, a custom-built instrument was used to measure conductivity under several stretching cycles. Samples were prepared as 3 × 1 cm; while one end was fixed, the other was engaged to a motorized stage. The samples were then stretched up to 500% of their original length and were relaxed at the rate of 0.005 ms^−1^. During stretching, resistance was measured using an Agilent 4263B LCR meter.

In addition to experimental characterization, a computational analysis evaluated different weight ratio percentages for their current density and electric field distribution during transcutaneous stimulation.

### 2.5. Electrode Array Fabrication

**Figure 1:**
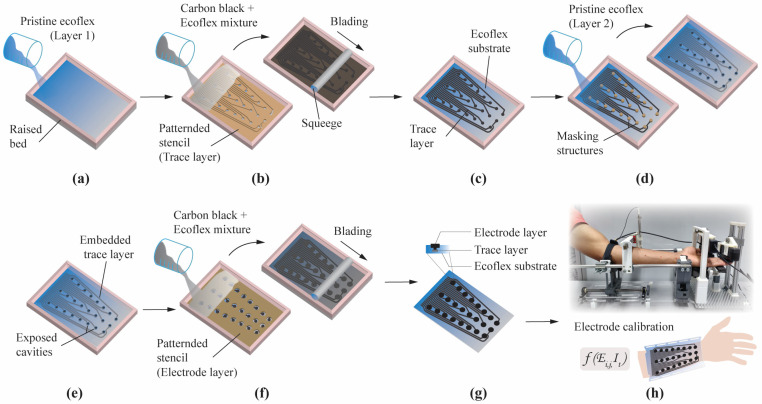
Multilayered screen printing of an electrode array. (**a**) Pristine Ecoflex forming the substrate layer. (**b**) The trace layer was screen-printed over the substrate layer using a patterned stencil. Here the base mixture (carbon black and Ecoflex) was evenly poured and smeared using a squeegee. (**c**) Exposed trace layer over the substrate layer. (**d**) 3D-printed masking structures were used to create cavities, while another layer of pristine Ecoflex concealed the trace layer. (**e**) Trace layer concealed by the second layer of pristine Ecoflex with cavities exposed. (**f**) The last layer, i.e., the electrode layer, was screen printed using a patterned stencil. The base mixture was again poured and smeared to fill the exposed cavities from the previous step. This process also bridged the electrodes to their respective traces in the trace layer. (**g**) Wearable sleeve array with exposed electrode layer. Insert shows the cross-section of an individual electrode. (**h**) Experimental setup to evaluate hand function during transcutaneous stimulation. Figure depicts initial scanning for motor point locations. Activating respective elements in the array ($E_{i,j}$) with appropriate stimulation waveform ($I_{t}$ ) elicited the desired outcome.

The screen-printing process for the electrode array followed the deposition of the base mixture over a substrate through patterns on a stencil, which formed desired patterns after curing [22,25]. In this study, this process was performed in multiple stages to include patterns of both stimulation electrodes and traces. Here, the traces were embedded into the sleeve, leaving only the electrodes exposed to the skin surface. Figure 1 illustrates the stages involved in the fabrication process.

Firstly, pristine Ecoflex was prepared by mixing 1A:1B by weight. Once degassed, it was poured into a mold and allowed to cool on a level surface at room temperature for nearly four hours. This cured Ecoflex formed the substrate layer of the sleeve. Secondly, the trace layer was prepared using the base mixture (CB and Ecoflex). A patterned stencil (trace layer) was placed on the cured substrate, and the base mixture was poured and smeared using a squeegee. It covered the entire pattern of the stencil, and the squeegee was daubed in one direction to yield good-quality patterning. The desired patterns formed the traces for the electrode upon curing. The third layer was another pristine Ecoflex layer that concealed the trace layer. Here, cavities were created using 3D-printed masking structures. The purpose of the cavities was to interconnect the trace layer to the electrode layer. This second layer of pristine Ecoflex concealed the trace layer with exposed cavities. Using a patterned stencil (electrode layer), the base mixture was evenly poured and smeared to fill up the exposed cavities from the previous step and formed the electrodes.

### 2.6. Experimental Validation

The validity of the electrode array to stimulate the forearm muscles was evaluated in healthy participants. Experimental procedures were conducted on five healthy participants (4M + 1F, 29.40 ± 2.28 yrs, BMI 25.44 ± 1.80) who had no contraindications to electrical stimulation. The University of Auckland Human Participants Ethics Committee approved this study. All participants gave informed consent. External stimulation was administered by a current-controlled stimulator, RehaStim^™^ 2 (Hasomed GmBH, Magdeburg, Germany), using a LabVIEW interface [26]. The stimulation pulse width (300 μs) and frequency (50 Hz) were kept constant. Wrist contraction was achieved by varying the electrode location ($E_{i,j}$) and stimulation amplitude (0–20 mA).

## 3. Results

### 3.1. Electrode Array Design

**Figure 2:**
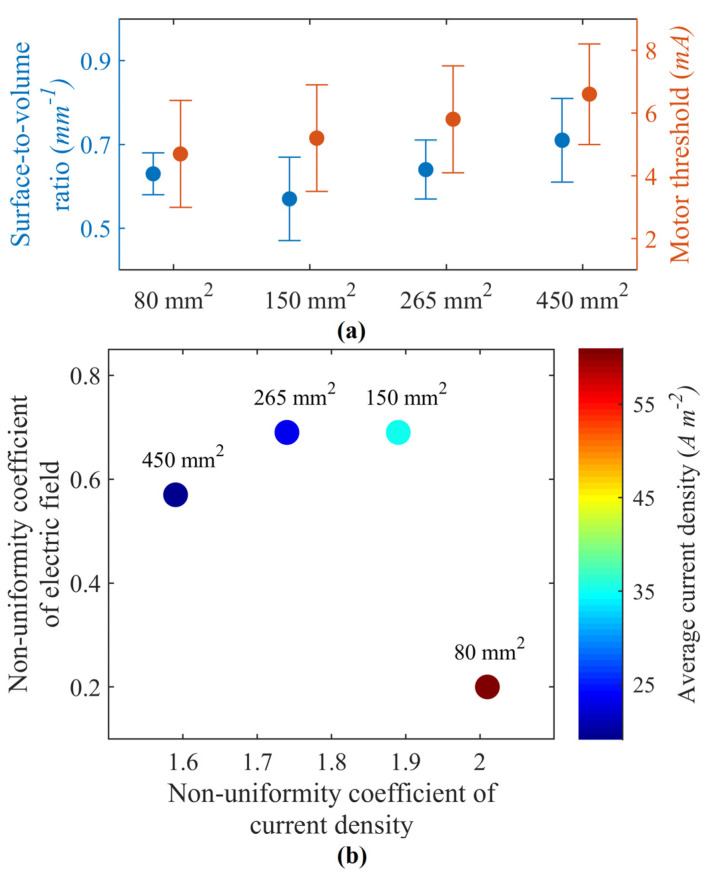
(**a**) Motor thresholds and normalized surface-to-volume ratios for respective activation volume. The error bars represent values for motor nerve stimulation at depths of 12.4, 14.6, and 16.8 mm. (**b**) Plot comparing model-predicted nonuniformity coefficients of current density and electric fields for electrodes with different surface areas. The color bar represents the average current density.

For simplicity of fabrication, circular-shaped electrodes with a thickness of 1.5 mm were considered. Four surface areas of 80, 150, 265, and 450 mm^2^ were analyzed, as these are widely used surface areas for transcutaneous electrode design. Both experimental and model-based analyses identified 150 mm^2^ as the suitable electrode size with the best trade-off performance for selectivity and comfortability. Figure 2a reports the SA:V ratios for various surface areas as a measure of selectivity. Here, the electrode with 150 mm^2^ had the highest selectivity and a lower motor threshold than the 265 and 450 mm^2^ electrodes. Comparing the current and field distributions, as in Figure 2b, the electrode with the smallest surface area, 80 mm^2^, had a high nonuniformity coefficient and a high magnitude of current density. At the same time, other electrodes had relatively similar profiles.

In addition, the electrode-array-based sleeve had 24 (8 × 3) individual electrodes. It was 256 mm long and had a trapezoidal shape to cover the forearm surface (width at the shorter end of 125 mm and at the longer end of 165 mm). While the distance between individual electrodes varied between 30 and 60 mm, for wrist flexion, electrode pairs that were 80 mm apart were chosen to elicit higher contraction levels.

### 3.2. Electrical Properties

**Figure 3:**
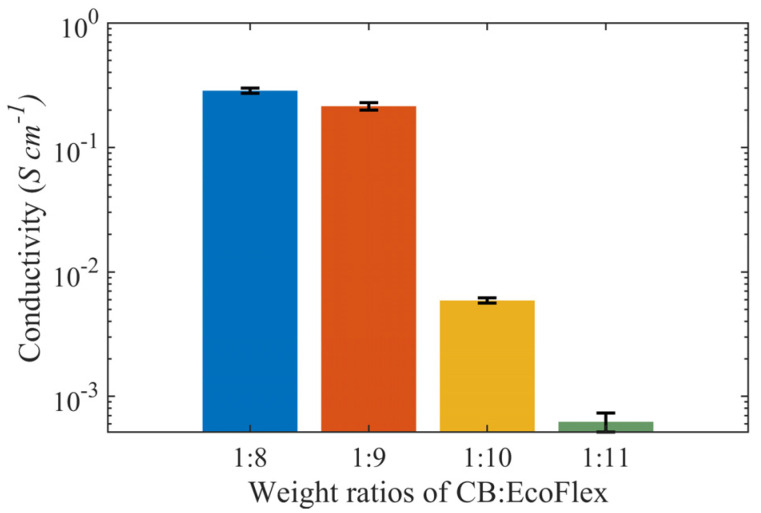
Conductivity measurements across sixteen samples of CB:Ecoflex grouped by different weight ratio percentages.

Four samples each for the weight ratio percentages of 1:8, 1:9, 1:10, and 1:11 were prepared to characterize their electrical conductivity. Figure 3 shows the conductivity trends for all 16 samples grouped by weight ratios. An apparent decrease in conductivity was observed with increased Ecoflex concentration. The conductivity gain was due to the addition of CB. Samples with a weight ratio percentage of 1:8 had the highest conductivity at 2.86 × 10^−3^ S cm^−^^1^, with a decreasing trend, and samples with a weight ratio percentage of 1:11 had the lowest conductivity of 6.23 × 10^−6^ S cm^−1^. Notably, samples with ratios of 1:8 and 1:9 had similar conductivities between 2 × 10^−3^ and 3 × 10^−3^ S cm^−1^.

**Figure 4:**
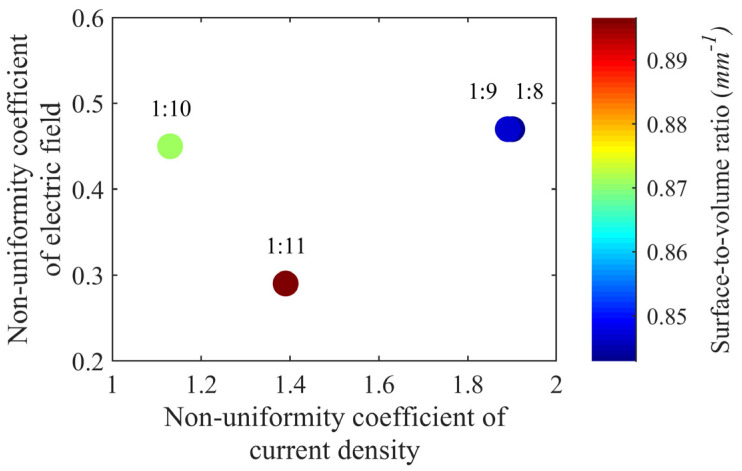
Comparison of model-predicted nonuniformity coefficients of current density and electric fields for electrodes with different weight ratio percentages. The color bar represents normalized surface-to-volume ratio.

Suitable conductivity levels that elicited selective and comfortable stimulation were identified using a model-based analysis [12]. Here, all four weight ratio percentages with thicknesses of 0.5, 1.0, and 1.5 mm were analyzed (Figure 4). To standardize the analysis, all twelve combinations had fixed stimulation parameters (10 mA, 300 µs), surface area (150 mm^2^), and interelectrode distance (80 mm). Here, the SA:V ratio was lower for 1:8 and 1:9 electrodes, implying comparative higher selectivity. Although the SA:V ratio was highest for the 1:11 electrodes, they had the smallest nonuniformity coefficients of current density and electric field.

**Figure 5:**
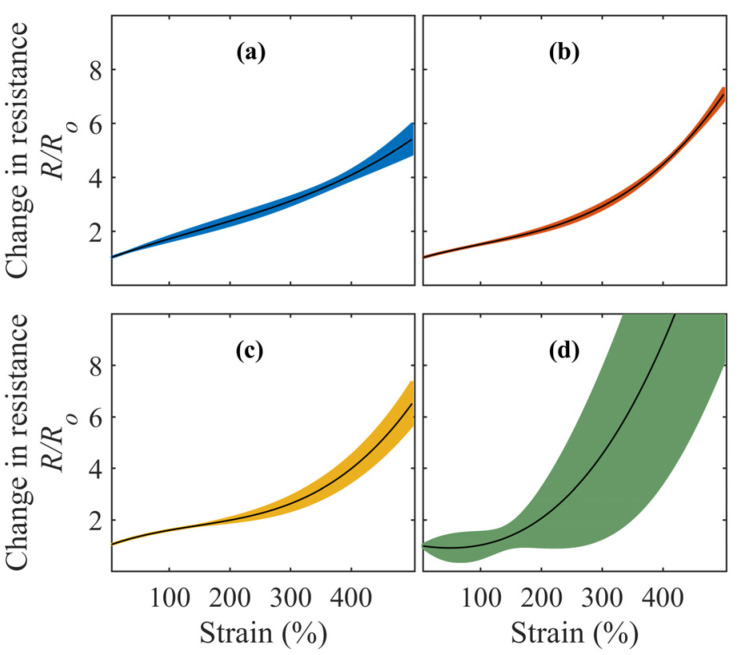
Change in resistance observed across twelve samples of CB:Ecoflex grouped by different weight ratio percentages of 1:8 (**a**), 1:9 (**b**), 1:10 (**c**), and 1:11 (**d**). Resistance changes were reported while the samples were subjected to three stretching cycles.

This study aimed to develop a conformable electrode-array-based sleeve. Thus, the conductive performance of these electrodes under cyclic stretching was assessed. Accordingly, Figure 5 compares the change in resistance for the weight ratios when stretched up to 500% of their original length. Three different samples were prepared for each weight ratio. The plots represent the averaged values across three separate stretching cycles performed on these three different samples. The change in resistance was taken as the ratio of resistance at the current strain level to the resistance at resting length.

A comparatively similar profile was observed across all the samples. Herein, the change in resistance increased with strain levels. With a factor of 4.5, the change in resistance was the lowest for the 1:8 electrode. The weight ratios of 1:9 and 1:10 had factors of 5.7 and 7.8, respectively. However, the 1:11 ratio exhibited drastic changes in resistance across the samples. At a stretch level of 400%, the change in resistance was 7.3, and it worsened to 26.6 when stretched to 494% of its original length. CB infused within elastomers tends to suffer from discontinuities during curing processes or homogenization. Thus, it was inferred from this weight ratio that the integrity of bonding between CB and the elastomer was greatly affected at higher strain levels.

### 3.3. Surface Characteristics

**Figure 6:**
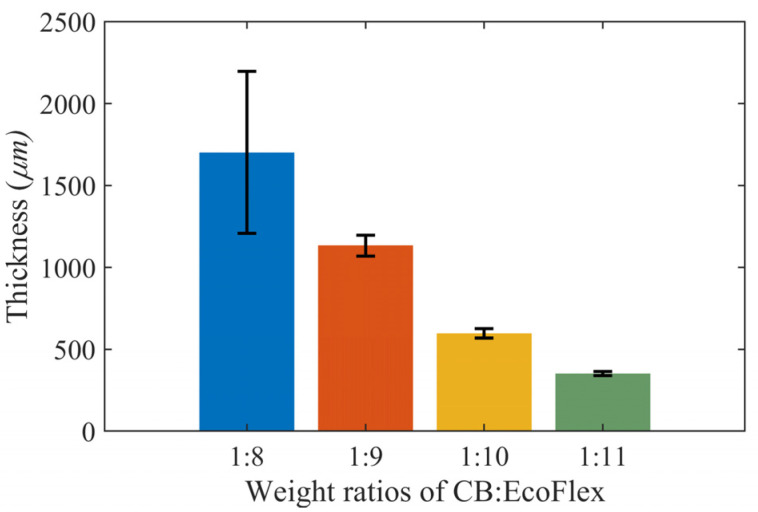
Average surface thickness across twelve samples of CB:Ecoflex grouped by different weight ratio percentages.

Stylus profilometry was used to assess the surface morphology of samples with different weight ratios. These samples were 0.1 mm thick and prepared using a stencil. As in Figure 6, the surface roughness increased with the concentration of CB. Accordingly, samples with a 1:8 weight ratio had a higher roughness with large variations at 1314.54 ± 712.21 µm. For samples with a 1:11 weight ratio, a smaller thickness of 351.62 ± 12.21 µm was observed. Nevertheless, similar surface profiles were observed for the 1:8 and 1:9 weight ratios based on thicknesses of 1314.54 ± 712.21 µm and 1132.83 ± 62.71 µm, respectively.

The mean surface profiles for the weight ratios of 1:8 and 1:9 were very similar, with thicknesses of 1314.54 ± 712.21 µm and 1132.83 ± 62.71 µm, respectively. However, the weight ratio of 1:8 had a higher deviation in thickness across the profiling length when compared to the sample with the 1:9 weight ratio. In addition to average surface thickness, surface roughness along the profiling length was observed across the samples. Similarly, the size of the peaks and troughs was large for samples with a 1:8 weight ratio. It was comparatively uniform with the 1:11 weight ratio.

**Figure 7:**
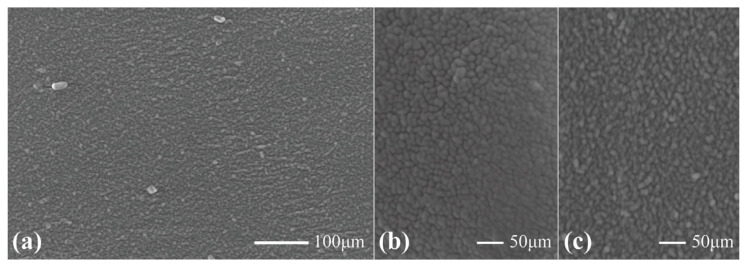
Scanning electron microscope images for 1:8 and 1:9 weight ratios of CB:Ecoflex. Images for 1:8 weight ratio magnified at ×250 (**a**) and ×450 (**b**), and 1:9 weight ratio magnified at ×450 (**c**).

Figure 7 exhibits the surface morphology of a sample with a ratio of 1:8 CB:Ecoflex observed using an SEM. Visually, the surface of the sample was comparatively rough (×200 magnification, Figure 7a), which was in accordance with the Stylus profilometry. With further magnification (×450, Figure 7b), discernible particles can be seen, leaving large peaks and troughs.

The 1:8 weight ratio had the lowest change in resistance under cyclic stretching. This was a critical design requirement to realize a conformable electrode-array-based sleeve.

### 3.4. Fabrication and Testing

Based on the characterization studies, the weight ratios of 1:8 and 1:19 were considered suitable for fabrication.

**Figure 8:**
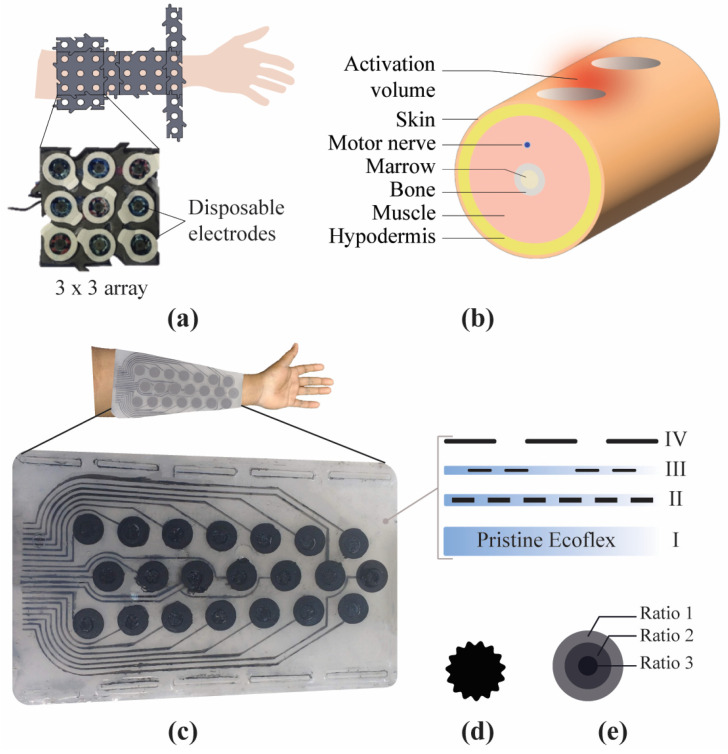
(**a**) Previous attempt of an electrode-array-based sleeve with disposable electrodes having a bulky construction. (**b**) Computational model of a forearm under transcutaneous stimulation. (**c**) Electrode-array-based sleeve fabricated using multilayered screen-printing process (in this study). (**d**,**e**) demonstrate that the fabrication process can allow for customizing the electrode elements in terms of shape and different materials.

Figure 8a shows an electrode-array-based sleeve fabricated using the multilayered screen-printing process. The insert shows a cross-section of the sleeve, which reveals several layers, including the pristine Ecoflex layer (I) that served as the substrate and provided structural support to the sleeve. This layer was 2 mm thick; the other layers (II, III, and IV) were 1.0 mm thick.

Following fabrication, the validity of the electrode array to stimulate the forearm muscles was assessed in healthy participants. With constant stimulation parameters (300 µs, 50 Hz), successful wrist contractions were elicited by varying the stimulation amplitude to 9.60 ± 1.36 mA and electrode locations.

## 4. Discussion

Electrode arrays can simplify user integration and its utility by allowing for easy recalibration and through customized stimulation delivery [2,27,28,29,30]. Nevertheless, user acceptance, ease of use, customizability, stimulation performance, and cost-effectiveness govern the translation of such systems from research-based to home-based settings. In an effort to address these factors, this study demonstrated the viability of fabricating a conformable electrode-array-based sleeve. The chosen multilayered screen-printing technique facilitated the holistic fabrication of stimulation electrodes and their traces using the same base material. Highly customizable electrode-array-based sleeves could be realized with a relatively cost-effective fabrication process.

### 4.1. Carbon-Infused Silicone-Based Elastomers

Here, the primary design requirement for the electrode array was for the sleeve, stimulation electrodes, and traces to be conformable. Thus, the sleeve could adapt to forearm movements. Additionally, the stimulation electrodes within the sleeve must have a recommended conductivity suitable for transcutaneous stimulation.

Thus, a silicone-based elastomer was used to make the electrodes inherently stretchable. However, these elastomers have poor electrical properties. Adding functional materials can alter the conductivity of silicone-based elastomers. Accordingly, the conductive properties of a silicone-based elastomer, EcoflexTM00-30, were modified by adding CB. The ratio of CB to Ecoflex^TM^ influenced the conductivity and printability of the composite. Different weight ratios of the base mixture were characterized for conductivity and surface morphology, and the weight ratio of 1:8 was identified to have optimal stimulation performance and was later used to fabricate a stretchable electrode-array-based sleeve.

Here, Ecoflex^TM^ 00-30 was the choice of material for its stretchability and biocompatibility. As a commercially available material, Ecoflex has been used for a wide range of applications in sensing and actuation [31,32]. Moreover, it is easy to work with and relatively inexpensive. In addition, the softness and stickiness of Ecoflex^TM^ can enable prolonged use. The addition of CB altered the conductive properties of Ecoflex while retaining its stretchability. Compared to other carbon-based materials, CB has been extensively researched [33,34,35], and its characteristics are well-known [23]. Moreover, it is inexpensive and can easily be dispensed into other materials without pretreatment.

Different weight ratios of CB and Ecoflex were characterized for conductivity and surface morphology. This study considered weight ratios of 1:8, 1:9, 1:10, and 1:11, which were chosen based on preliminary experimentation considering the dispersion of CB and the expected conductivity. Among them, the weight ratios 1:8 and 1:9 had the desired properties. The literature on transcutaneous stimulation electrodes has limited reporting on ideal electrical properties. Nevertheless, the resistivity range for commercial stratified electrodes varies between 30 and 150 Ωm [24], and textile electrodes have a resistivity between 70 and 160 Ωm [14]. In addition, textile electrodes with a low resistivity of 0.714 Ωm were reported [9]. In this study, the resistivity of the 1:8 and 1:9 electrodes varied between 3.33 and 4.76 Ωm. These resistivities adequately elicited muscle contraction at comfortable levels. Moreover, graphene-ink-coated stimulation electrodes with a reported sheet resistance of 903.51 ± 262.15 $Ω/{□}^{-1}$ (3 mm thickness) had similar properties to this study [15].

In addition, it was vital to assess the resistance of the stimulation electrodes under stretching. The least change in resistance while stretching was observed for the 1:8 weight ratio. Additionally, the problem of discontinuities during the curing process or homogenization was noticed among samples with a higher weight ratio of Ecoflex (1:11). Due to this effect, drastic changes in resistance values were observed, and stimulation delivered via such electrodes could have adverse effects of stimulation-induced burns or discomfort. While adding or removing CB altered the surface morphology, the surface was comparatively rough for the 1:8 weight ratio. However, this can be leveraged, as the uneven surface may favor improved electrode–skin adhesion. Considering the above factors, the sleeve and traces were fabricated with a 1:8 weight ratio of CB and Ecoflex to derive the desired conformable electrode-array-based sleeve. Nevertheless, long-life wearable sleeves with improved stimulation performance can be realized using the improved design principles [31] and by further optimizing the weight ratios and other functional properties of the base materials.

### 4.2. Screen Printing of Electrode Arrays

Figure 1 shows that several layers of CB:Ecoflex and pristine Ecoflex were bonded using the multilayer screen-printing process. Compared to other fabrication techniques, screen printing is widely used for being cost-effective and straightforward [16,21,34,35]. Intricate patterns can be made relatively simple with masking stencils and a squeegee. Here, the patterned materials were kept under higher-than-normal temperatures to accelerate the curing process. However, it could also be cured at a normal room temperature, which obviates the need for temperature-controlled ovens. In addition, both the electrodes and the traces were fabricated using a single fabrication process. The fabrication method was simple, applicable for large-scale production, highly customizable, and could support various materials. In addition, the fabrication base materials that were considered here, including CB and Ecoflex, were cost-effective.

Previous attempts on stretchable fabric for electrotherapy have been proposed [36]; however, they have not been implemented for electrode-array-based stimulation. Moreover, electrode arrays fabricated through screen printing [16] and garment-based procedures [9] have been proposed. Although they have been able to demonstrate flexible designs, the work here is the first of its kind to develop a stretchable (conformable) array that included cost-effective materials and a fabrication technique that coupled the production of electrode and trace elements in a single fabrication step.

### 4.3. Conformable Electrode Arrays

This paper presented the rationale, design, and proof of concept for a conformable, wearable electrode-array-based sleeve. In addition to challenges in choosing the appropriate material for the stimulation electrodes, considering optimal designs and materials for the traces and connections was equally important [37]. The conformability of electrodes is very important for user comfort and when compensating for the displacement of motor points following forearm rotation. Moreover, a previous study on the displacement of motor points under forearm rotation showed that the stimulation of motor points could only displace up to 100% [5]. The reason for considering 500% strain in this study was to improve the durability of the electrode-array-based sleeve, as it may be exposed to several cycles of putting on and taking off. Still, the weight ratios of 1:8 and 1:9 had integral performances far above the physiologically valid displacement.

### 4.4. Advent of Personalizable Electrode Arrays

To encourage prolonged use, electrode arrays must be low-profile and comfortable. Likewise, they can dynamically adapt to choose a specific electrode in an array to acquire the best muscle response, allowing a nonexpert to operate the device effectively in a home environment. Considering these factors can improve the usability of electrode arrays, which, in turn, promotes their utility for home-based rehabilitation. Influencing the electrode performance by altering the electrode geometry can result in highly personalized, easy-to-fabricate, and cost-effective electrodes for wearable neuroprostheses. Moreover, when combined with advances in stimulation techniques, electrode configurations, material properties, and interface layers, the performance of these electrodes can be further boosted. This idea can be extended to whole-body garment-based designs [8].

Several subject-specific factors influence stimulation performance; hence, these electrodes can be tailored to individuals. The applicability of electrode arrays can be improved by considering designs that can be derived from the shape and size of the stimulation zones [38]. Most importantly, these electrodes should be flexible and stretchable and should simultaneously maintain conductivity; thus, comfortable stimulation can be delivered. Considering the above factors, electrode arrays fabricated using screen printing offered stretchability; they were lightweight, and the fabrication process facilitated rapidly customizable electrode designs. Furthermore, the use of Ecoflex made the electrode array biocompatible. Given the rapid fabricability of the electrodes, they could be altered to fit any forearm. Additionally, the sleeve designs could be improved to accommodate breathability.

Moreover, CB-based Ecoflex sensors have been proposed for sensing EMG or biopotential recordings [39]. As it is important to know the direction of the deformation that a sleeve undergoes, the change in resistance during stretching can be used to sense the direction of shear, thus avoiding the need for external sensors. Then, further calibration can adjust the stimulation parameters or site of stimulation using automated algorithms [5] or kinematic feedback [40].

Uniform current distribution on an electrode surface is essential to avoid hot spots that may cause discomfort and pain [14]. Although the 1:8 electrodes were preferred, they had high nonuniformity coefficients compared to the 1:11 electrodes. The performance of these electrodes could be improved by modifying the electrode surface or adding current redistribution layers.

While this study demonstrated the feasibility of fabricating electrode arrays using a multilayered screen-printing technique, in future work, we aim to validate these electrode arrays further to selectively stimulate forearm muscles and compare their performance with commercial electrodes. Stimulation outcomes can be evaluated using dynamometry (muscle contractions) and VAS scores (comfort) [12]. As proposed in Figure 8d, the feasibility of fabricating conductive layers with radially varying impedance can be explored to improve stimulation performance using a multilayered screen-printing technique. Furthermore, integrated strain sensors can aid with autocalibration tasks for electrode repositioning.

## 5. Conclusions

The viability of fabricating conductive layers infused with silicone-based elastomers was demonstrated using a multilayered screen-printing technique. Herein, the conductivity of a silicone-based elastomer was altered by the addition of CB. The resistivity, stretchability, and surface morphology of different weight ratios of CB and Ecoflex^TM^ were assessed. An optimal ratio of CB infused within the elastomer that tended to maintain the stimulation performance after several stretching cycles was identified and later used to fabricate an electrode array. In addition to fabricating a stretchable electrode-array-based sleeve, the multilayered screen-printing technique also facilitated the holistic fabrication of stimulation electrodes and their traces using the same base material, which was a straightforward fabrication process. Functional electrical stimulation through transcutaneous electrodes has several clinical applications, including functional training (motor recovery), muscle strengthening, and pain relief. These applications can greatly benefit from the proposed electrodes. Furthermore, the personalization offered by these electrodes encourages the realization of comfortable, wearable, and cost-effective neuroprostheses.

## Data Availability

Not applicable.
